# Lovastatin lactone may improve irritable bowel syndrome with constipation (IBS-C) by inhibiting enzymes in the archaeal methanogenesis pathway

**DOI:** 10.12688/f1000research.8406.3

**Published:** 2016-06-22

**Authors:** Steven M. Muskal, Joe Sliman, John Kokai-Kun, Mark Pimentel, Vince Wacher, Klaus Gottlieb

**Affiliations:** 1 Eidogen-Sertanty, Oceanside, CA, USA; 2 Synthetic Biologics, Rockville, MD, USA; 3 Cedars-Sinai Medical Center, Los Angeles, CA, USA

**Keywords:** IBS, IBS-C, Lovastatin, homology modeling, multi-site docking

## Abstract

Methane produced by the methanoarchaeon *Methanobrevibacter smithii* ( *M. smithii*) has been linked to constipation, irritable bowel syndrome with constipation (IBS-C), and obesity. Lovastatin, which demonstrates a cholesterol-lowering effect by the inhibition of HMG-CoA reductase, may also have an anti-methanogenesis effect through direct inhibition of enzymes in the archaeal methanogenesis pathway. We conducted protein-ligand docking experiments to evaluate this possibility. Results are consistent with recent clinical findings.

METHODS: F420-dependent methylenetetrahydromethanopterin dehydrogenase ( *mtd*), a key methanogenesis enzyme was modeled for two different methanogenic archaea: *M. smithii* and *Methanopyrus kandleri*. Once protein models were developed, ligand-binding sites were identified. Multiple ligands and their respective protonation, isomeric and tautomeric representations were docked into each site, including F420-coenzyme (natural ligand), lactone and β-hydroxyacid forms of lovastatin and simvastatin, and other co-complexed ligands found in related crystal structures.

RESULTS: 1) Generally, for each modeled site the lactone form of the statins had more favorable site interactions compared to F420; 2) The statin lactone forms generally had the most favorable docking scores, even relative to the native template PDB ligands; and 3) The statin β-hydroxyacid forms had less favorable docking scores, typically scoring in the middle with some of the F420 tautomeric forms. Consistent with these computational results were those from a recent phase II clinical trial ( NCT02495623) with a proprietary, modified-release lovastatin-lactone (SYN-010) in patients with IBS-C, which showed a reduction in symptoms and breath methane levels, compared to placebo.

CONCLUSION: The lactone form of lovastatin exhibits preferential binding over the native-F420 coenzyme ligand *in silico* and thus could inhibit the activity of the key *M. smithii* methanogenesis enzyme *mtd* *in vivo*. Statin lactones may thus exert a methane-reducing effect that is distinct from cholesterol lowering activity, which requires HMGR inhibition by statin β-hydroxyacid forms.

## Introduction

Irritable bowel syndrome (IBS) affects as many as 45 million people in the United States, and up to 23% of the worldwide population ^1^. Depending on the region, as many as 43.3% of these patients will have irritable bowel syndrome with constipation (IBS-C) ^2^. The illness affects both men and women; however, two-thirds of diagnosed sufferers are women. Studies have linked methane production to the pathogenesis of constipation and IBS, as well as obesity ^3^. Methanogens – i.e. anaerobes that respire hydrogen to produce methane - are found in many habitats supporting anaerobic biodegradation of organic compounds, including human and animal intestinal tracts ^4, 5^. Archaea are the only confirmed, naturally occurring biological sources of methane. *Methanobrevibacter smithii* ( *M. smithii*) is the predominant methanogen in the human intestine accounting for 94% of the methanogen population ^3^.

The isoprenoid biosynthesis for the main cell membrane components in archaea (archaeol) relies on the same enzyme that catalyzes the biosynthesis of the isoprenoid cholesterol in humans - HMG-CoA reductase (mevalonate pathway) ^6^. It has been previously suggested that statins, i.e. known HMG-CoA reductase inhibitors, can also interfere with the biosynthesis of the archaeal cell membrane and thus inhibit archaeal growth ^7^. Statins, specifically lovastatin, have been shown to lower methanogenesis in human stool samples ^8^ and can inhibit archaeal cell membrane biosynthesis without affecting bacterial numbers as demonstrated in livestock and humans. Lovastatin is a secondary metabolite produced during fungal growth and is found in oyster mushrooms ^9^, red yeast rice ^10^, and Pu-erh ^11^.

Humans and archaea utilize the HMGR-I isoform for isoprenoid biosynthesis ^12^. Mevastatin and lovastatin were both shown to inhibit growth of several rumen Methanobrevibacter isolates in the ~10 nmol/ml range ^3^. While it is believed that statins inhibit methane production via their effect on cell membrane biosynthesis mediated by inhibition of HMG-CoA reductase, there is accumulating evidence for an alternative or additional mechanism of action where statins inhibit methanogenesis directly ^13^. In one case, *in silico* molecular docking of the methanogenic enzyme F420-dependent NADP oxidoreductase ( *fno*) showed that both lovastatin and mevastatin had higher affinities for the F420 binding site on *fno* than did F420 itself. It has been suggested that lovastatin may act as an inhibitor of *fno* ^14^.

Several reviews have appeared describing the reduction of CO _2_ to CH _4_ in methanoarchaea ^15^. Considering other mechanisms by which statins may inhibit methanogenesis directly, we have explored two important dehydrogenases in the main methanogenesis pathway, including F420-dependent methylenetetrahydromethanopterin dehydrogenase of *M. smithii* [ **A5UMI1** - 275 amino acid residues], and evolutionarily related F420-dependent methylenetetrahydromethanopterin (methylene-H(4)MPT) dehydrogenase ( *mtd*) of *Methanopyrus kandleri* [ **Q02394** – 358 amino acid residues]. Both only leverage F420 as a coenzyme, which assisted our computational analyses by avoiding issues associated with an NADP induced fit. The **Q02394** sequence does not have crystallographic structural information in the Protein Data Bank (PDB) ^16^, so we needed to identify acceptable templates to model this sequence. The **A5UMI1** sequence, however matched the 3IQZ co-complex with methylenetetrahydromethanopterin (H4M) having 52% sequence homology. While both sequences required modeling, we needed to identify one or more acceptable templates for **Q02394**. After modeling and receptor site identification, we docked and rank-ordered multiple ligand variations across several modeled receptor sites to evaluate preferential binding characteristics for the ligands in question.

## Methods

Protein sequences were extracted from UniProt ^17^. Many protein structure modeling methods have been developed and are available with most performing well given crystallographic template(s) sharing sufficient sequence homology with target sequences of interest ^18^. The Eidogen StructFast ^19, 20^ technology is well suited for this type of modeling. StructFast can operate in an automated mode where the best PDB template is automatically selected, or in a directed mode where modeling is guided based on a suggested PDB template ^21, 22^.

Once models for **A5UMI1 and Q02394** were developed with StructFast, ligand binding sites were identified by inference from the respective PDB templates used in modeling and from the Eidogen SiteSeeker algorithm ^23^. SiteSeeker looks for concave, surface features sufficiently exposed to enable ligand binding while also considering evolutionary conservation of sequence. In addition to sites identified by SiteSeeker, other sites were manually inferred within PyMOL v1.8 after aligning models and templates containing their respective co-complexed ligands. Residues on model structures with a 7Å cutoff of co-complexed ligands within the templates were exported and also processed as sites.

Ligands were carefully prepared considering different protonation states, isomers, and tautomers. We standardized charges, added missing hydrogens, enumerated ionization states, ionized functional groups, generated tautomers and isomers, and generated starting-point 3D coordinates for each ligand using BIOVIA’s (Accelrys’) Pipeline Pilot technology v8.5 ^24^. Ligands were finally prepared into mol2 format ^25^. Each representation was then docked into each identified site and scored using AutoDock Vina v1.1.2 ^26^, an open docking technology that utilizes grid-based energy evaluation and efficient search of ligand torsional freedom. AutoDock Vina has been tested against the Directory of Useful Decoys, performing frequently better than many commercially available docking programs.

The AutoDock Vina system requires that receptor site files be formatted in the PDBQT [Protein Data Bank, Partial Charge (Q), & Atom Type (T)] molecular structure file format. The MGLTools v1.5.4 ^27^ were used for this file format conversion. Additionally, AutoDock Vina requires a defined grid box surrounding the receptor site residues. Here, we identified the center of mass of each receptor site using all atoms in the receptor site PDB file. We then calculated within Pipeline Pilot the maximum distance between any atom in the receptor site and the centroid in each x,y,z-direction. The lengths of each grid box were configured with these maximums. To insure reproducibility and comparability of docking simulations, we initiated each AutoDock Vina run with the same random seed value of 1162467901.

## Results and discussion

Raw data for ‘Lovastatin lactone may improve irritable bowel syndrome with constipation (IBS-C) by inhibiting enzymes in the archaeal methanogenesis pathway’ Includes developed models (pdb format), ligands (mol2 format), sites (pdb format), and vina config files Click here for additional data file. Copyright: © 2016 Muskal SM et al. 2016 Data associated with the article are available under the terms of the Creative Commons Zero "No rights reserved" data waiver (CC0 1.0 Public domain dedication).

Lovastatin-lactone v. F420 in the A5UMI1 site. The Lovastatin-lactone form is shown with green sticks, and F420 with red sticks. Residues within 5 angstroms of the ligands are labeled and highlighted. Hydrophilic site residues are shown in cyan, and hydrophobic residues in grey. Click here for additional data file. Copyright: © 2016 Muskal SM et al. 2016 Data associated with the article are available under the terms of the Creative Commons Zero "No rights reserved" data waiver (CC0 1.0 Public domain dedication).

### Protein modeling and site identification

We identified three different PDB templates that had sufficient sequence homology to model the **Q02394** sequence, by identifying other PDB co-complexes containing ligands with high 2D similarity to the H4M ligand. The top three PDBs showing significant sequence homology to **Q02394** included: 3F47 (57%), 3H65 (57%), and 4JJF (52%). Each template was used to model **Q02394.** In each case, ligand-binding sites were readily inferred from the ligand binding sites found in the respective template structures.

The modeling of sequence **A5UMI1** was straightforward given its high 52% sequence homology to 3IQZ. Other templates (e.g. 1U6I, 1U6J, 1U6K, and 1QV9) were possibilities, but each had slightly lower resolutions, earlier deposit dates, and/or were in apo form. Each 3IQZ chain (A-F) was considered, given the possibility that one template-chain might offer additional or different insight into possible ligand binding locations. The Eidogen SiteSeeker algorithm identified only one site when template chains A, C, D were used, while two sites were identified in models leveraging template chains B, E, F. Unfortunately, the H4M site from the 3IQZ template was not easily inferred into any of the **A5UMI1**/3IQZ-based models, because 3IQZ has multiple chains involved in H4M binding.

**Figure 1.**
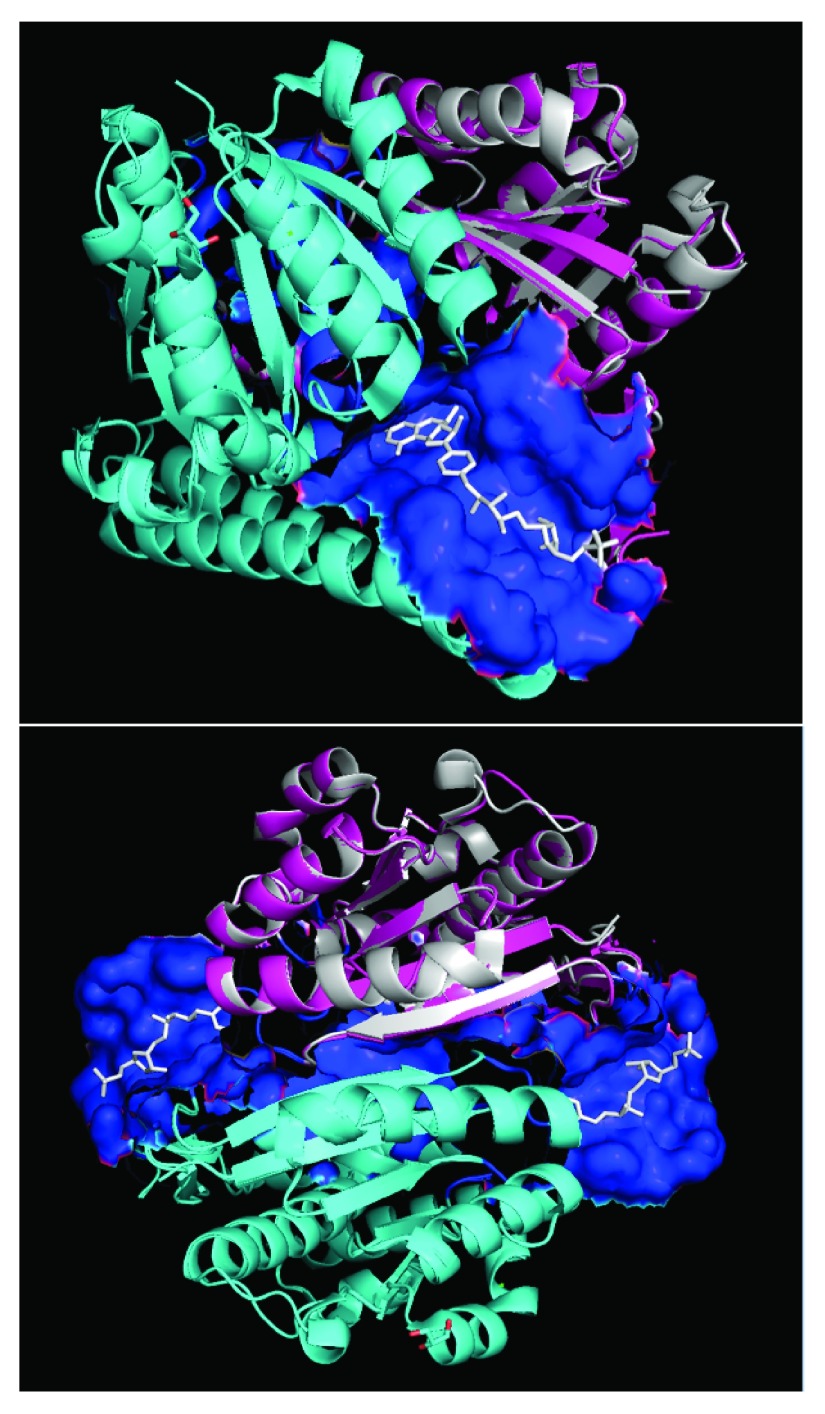
Modeled quaternary structure of **A5UMI1**/3IQZB (cyan) and **A5UMI1**/3IQZF (pink) after respective alignments onto chain-B and chain-F of 3IQZ within PyMOL ^28^. 3IQZ’s chain-F is highlighted in silver. Dual chain model site residues (blue surface) were inferred from residues in chain-B and chain-F models that are within 7 Å of the 3IQZ ligand (H4M - white). 3IQZ’s chain-B and chain-F form a quaternary structure with two different H4M binding sites (bottom).

Modeling sequences from PDB templates is done with individual chains. Quaternary modeling using models of individual chains can be very challenging. We manually modeled the H4M site as described in Figure 1. Since our aim was to dock all ligands across all possible ligand binding sites, we included the sites identified by inference (i.e. where ligands were present in templates), by the SiteSeeker algorithm run across single chain models, and by manually modeled sites as described by Figure 1. A total of 10 ligand-binding sites ( Table 1) were identified across all the **Q02394** and **A5UMI1** models.

**Table 1.** Ligand binding sites identified and inferred from models. Four sites from the **A5UMI1** modeling and six sites from **Q02394** modeling were used in the docking simulations.

A5UMI1	Q02394
3IQZB (H4M 7Å), 1 chain	3F47 (I2C)
3IQZB (SiteSeeker1)	3F47 (SiteSeeker)
3IQZB (SiteSeeker2)	3H65 (H4M)
3IQZB (H4M)_3IQZF (7Å)	3H65 (I2C)
	4JJF (FE9)
	4JJF (SiteSeeker)

### Ligand processing

The key ligands for this effort included lovastatin (lactone and hydroxyacid forms), F420, and simvastatin (lactone and hydroxyacid forms) and processed ligands that were found in the PDB templates used to model each sequence: 803, F42, H4M, I2C, FE9, SIM, 116, HMG, and 882. The latter four (SIM, 116, HMG, 882) were ligands found in the positive control templates for completeness.

The PDB often contains problematic ligand structures, so we processed both PDB ligands and ligands extracted from PubChem ^29^ for lovastatin (lactone and hydroxyacid forms), F420, and simvastatin (lactone and hydroxyacid forms). It should be noted, the PDB considers 803 as lovastatin (lactone form), F42 as coenzyme-F420, and SIM as simvastatin (hydroxyacid form), though their actual structural forms may vary depending on the PDB entry. This is why we also use PubChem structural representations for lovastatin, F420, and simvastatin.

It is well established that the β-hydroxyacid form and not the closed-ring lactone form of lovastatin is the active HMGR-binding form of the molecule ^30^. Simvastatin and lovastatin are commercially available in the lactone form; they behave as prodrugs which inhibit HMGR only after the opening of the lactone ring into the hydroxyacid form ^31, 32^. The degree of hydrophobicity of imidazole derivatives correlates with improved activity against human methanogenic archaea ^33^.

Each ligand was computationally processed in the same way prior to docking. BIOVIA’s (Accelrys’) Pipeline Pilot was used for this ligand preparation. First, stereochemistry and charges were standardized, then ionized at pH 7.4, then tautomers (if present) were enumerated, and finally initial 3D models were determined. AutoDock Vina explores ligand 3D conformation, so the initial 3D models were simple starting points. Additionally, ligands were processed without the above standardization, ionization, and tautomer exploration. Each ligand representation was considered in the docking runs. Ligands processed with the standardization sequenced contained the prefix “STD_,” and ligands without standardization contained the prefix “RAW_.” Together, these expanded ligand representations can help gauge the docking algorithm’s sensitivity to the ligand’s structural representation.

### Docking multi-ligand variations/multi-receptor sites

A total of 88 ligand variations were systematically docked into the 10 identified binding sites across all the **A5UMI1** and **Q02394** models for a total of 880 docking simulations. Even though AutoDock Vina achieves two orders of magnitude speed-up and significantly improves the accuracy of the binding mode predictions compared to AutoDock 4, 880 docking simulations could have taken several weeks to complete. To accelerate the effort, we requisitioned a compute cluster in the Amazon EC2 ^34^ cloud environment for approximately three days at a cost under $60.

The docking process scores ligand conformations based on ligand conformation and ligand-to-receptor interactions within a grid box. After the 880 docking simulations were complete, we rescored all docked ligand variations against their respective full model structures. This enabled a more realistic rank ordering given possible overlap with a docked ligand and other portions of a model not represented in the rectangular box. This also served as an internal control, since rescoring was completed independently of the docking simulations.

Since it is unknown which (if any site) might actually engage the ligands of interest, we calculated the average, minimum, and maximum affinity of each ligand/variation for each of the 10 sites. The top-two sites (highlighted in bold in Table 2) were used to then rank order each ligand. Table 3 shows the rank ordered ligands using the AutoDock Vina overall score, which considers steric interactions (Gauss 1, Gauss 2, and steric), dispersion/repulsion, hydrophobic interaction between hydrophobic atoms, and, where applicable, hydrogen bonding.

**Table 2.** Average, minimum, and maximum affinity for each site. Affinities were computed from AutoDock Vina ^26^. The top two scoring sites from **A5UMI1** and **Q02394** are in bold. These sites were used to rank the ligands in Table 3.

Docking Site	Average Affinity (kcal/mol)	Min Affinity (kcal/mol)	Max Affinity (kcal/mol)
**A5UMI1_3IQZB (SiteSeeker2)**	**-7.87**	**-9.01**	**-6.08**
**Q02394_4JJF (SiteSeeker)**	**-7.03**	**-9.07**	**3.88**
Q02394_3F47 (I2C)	-6.34	-9.34	24.92
A5UM1_3IQZB (H4M)_3IQZF (7Å)	-5.57	-8.56	6.29
Q02394_4JJF (FE9)	-0.22	-9.25	247.57
A5UMI1_3IQZB (H4M 7Å), 1 chain	0.18	-7.50	320.44
Q02394_3H65 (I2C)	0.80	-9.52	249.56
Q02394_3H65 (H4M)	3.90	-6.19	125.33
Q02394_3F47 (SiteSeeker)	168.88	-6.21	277.08
A5UMI1_3IQZB (SiteSeeker1)	214.07	-7.42	355.49

**Table 3.** Average AutoDock Vina scores over the top-two sites (see Table 2). Statin ligands highlighted in green are lactone form, or red if hydroxyacid form. F420 ligands are in blue. Tautomeric representations are included in each average. Standardized ligands are prefixed with “STD_,” those without standardization are prefixed with “RAW_” (see text). Ligand names have suffixes containing either the PDB entry they were originally extracted from, or their respective PubChem ^29^ CIDs.

Ligand	Average AutoDock Vina Score A5UMI1_3I QZB (SiteSeeker2) + Q02394_4 JJF (SiteSeeker)
RAW_803_1cqp	13.86
STD_803_1cqp	13.86
RAW_Simvastatin_pubchem_54454	14.34
STD_Simvastatin_pubchem_54454	14.34
RAW_Lovastatin_pubchem_53232	14.42
STD_Lovastatin_pubchem_53232	14.42
RAW_FE9_4jjfA	16.31
RAW_FE9_4yt4A	19.91
RAW_I2C_3f47A	22.35
STD_F42_3iqe	26.34
RAW_F42_3iqe	26.99
STD_SimvastatinAcid_pubchem_64718	27.23
STD_SIM_1hw9	27.66
STD_LovastatinAcid_pubchem_64727	27.92
RAW_SimvastatinAcid_pubchem_64718	29.52
RAW_LovastatinAcid_pubchem_64727	29.89
RAW_SIM_1hw9	30.68
STD_882_2q1l	33.31
RAW_882_2q1l	33.87
STD_116_1hwj	34.04
STD_H4M_1y60	39.54
RAW_116_1hwj	39.92
RAW_H4M_1y60	40.86
RAW_F42_3b4yA	54.70
STD_F420_pubchem_123996	56.39
RAW_H4M_3h65A_1607662	58.46
STD_F42_4qvb	61.33
RAW_F42_4qvb	62.72
RAW_F420_pubchem_123996	64.00
STD_F420_pubchem_123996	64.00
RAW_F42_1jayA	67.77
STD_F420_pubchem_123996	69.50
STD_H4M_3h65A	72.91
STD_HMG_1dq9	107.17
STD_FE9_4jjfA	111.50
STD_FE9_4yt4A	286.45
STD_F42_3b4yA	571.39
STD_F42_1jayA	835.97
RAW_HMG_1dq9	2671.39
STD_I2C_3f47A	12109.50

Given the rank ordering in Table 3, several observations became evident:

1) Consistent with Sharma *et al.* ^14^, the lactone form statins docked into each site with favorable site interactions (i.e. lower docking scores) as compared to F420 for the same sequence/site grouping.
2) The statin lactone forms generally had more favorable docking scores, even relative to the native template PDB ligands.
3) The statin hydroxyacid forms had less favorable docking scores and typically scored in the middle with some of the F420 forms.
4) The F420 scores were generally the lowest for each sequence/site models of **A5UM1** and **Q02394**.

**Figure 2.**
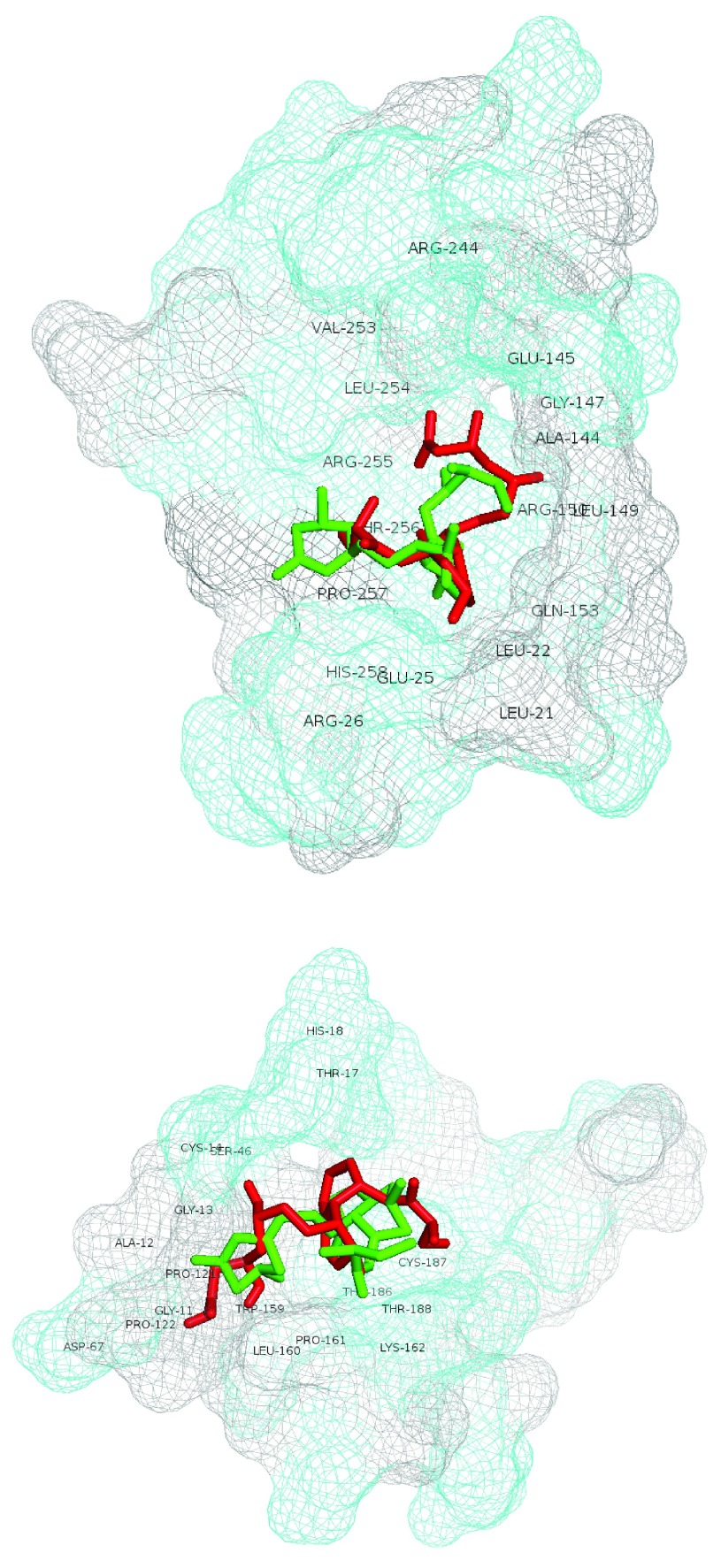
Best scoring lovastatin-lactone and -hydroxyacid poses in **A5UMI1** 3IQZB_SiteSeeker2 (top) and **Q02394** 4JJF_SiteSeeker (bottom). Lovastatin-lactone form is shown with green sticks and hydroxyacid form with red sticks. Residues within 5 angstroms of ligands are labeled. Hydrophilic site residues are shown in cyan and hydrophobic residues in gray.

Table 4 (a,b) details the AutoDock Vina scoring metrics of lovastatin-lactone v. lovastatin-hydroxyacid across the top two modeled sites. The lovastatin lactone form had better AutoDock scores across each site as compared to the hydroxyacid form. Similarly, the calculated affinity (kcal/mol) of the lactone form was better within both modeled **A5UMI1** sites. Figure 2 depicts the best scoring lovastatin-lactone and –hydroxyacid poses in the **A5UMI1** modeled site (top) and the **Q02394** modeled site (bottom). The **A5UMI1** modeled site is more spherically form fitting while the **Q02394** modeled site is more elongated. The **A5UMI1** site also contains a greater concentration of hydrophilic resides (depicted in cyan in Figure 2). In each modeled site, the best scoring lactone and hydroxyacid form were docked roughly in the same position with similar interactions, however the lactone form contained more favorable intermolecular feature.

**Table 4 (a,b).** Lovastatin-lactone (a) v. lovastatin-hydroxyacid (b) metrics across the top two modeled receptor sites. AutoDock4.1Score is a weighted sum of steric interactions (Gauss 1, Gauss 2, and steric), repulsion, hydrophobic interaction between hydrophobic atoms, and, where applicable, hydrogen bonding ^26^.

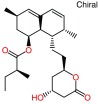		
a) Lovastatin (lactone): RAW_803_1cqp		
Site	A5UMI1 3IQZB (siteSeeker2)	Q02394 4JJF (siteSeeker)
Affinity (kcal/mol)	-7.2	-6.5
Gauss 1	54.5	66.1
Gauss 2	1273.8	1276.2
Repulsion	0.8	2.1
Hydrophobic	38.6	19.7
Hydrogen	2.1	2.6
AutoDock4.1 Score	14.3	13.4
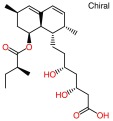		
b) Lovastatin (hydroxyacid): mevinolinic acid; PubChem 64727		
Site	A5UMI1 3IQZB (siteSeeker2)	Q02394 4JJF (siteSeeker)
Affinity (kcal/mol)	-6.9	-6.4
Gauss 1	78.8	77.6
Gauss 2	1360.5	1369.1
Repulsion	2.8	1.6
Hydrophobic	38.0	26.6
Hydrogen	5.3	2.9
AutoDock4.1 Score	28.2	31.6

**Figure 3.**
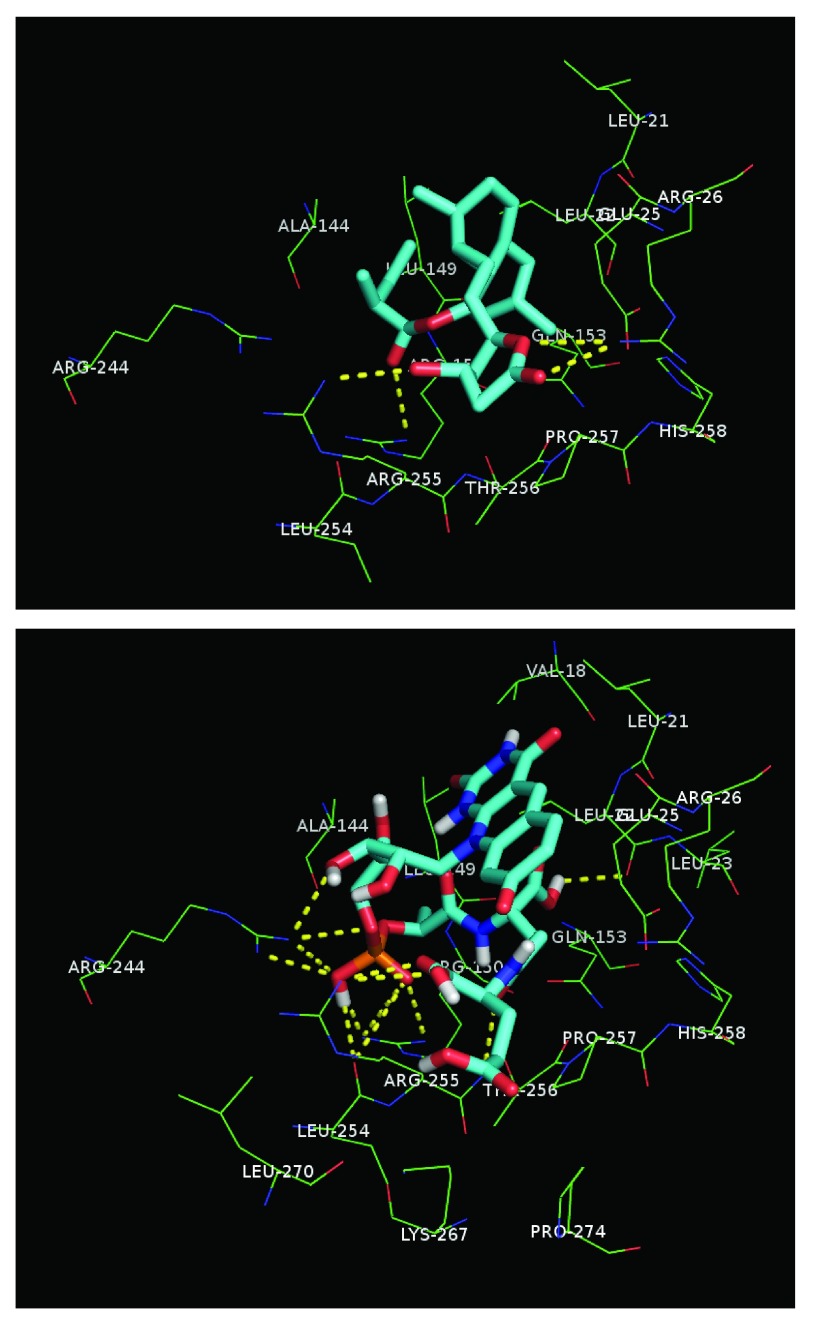
Lovastatin-lactone 1: (top) [Calculated affinity: -7.2 (kcal/mol); AutoDock4.1Score: 14.3]; 2: (bottom) F420 [Calculated affinity: -6.99 (kcal/mol); AutoDock4.1Score: 63.3] docked into A5UMI1_3IQZB_SiteSeeker2. Hydrogen bond interactions are denoted with yellow dotted lines.

Figure 3 depicts lovastatin-lactone (top) v. F420 (bottom) docked into the top **A5UMI1** modeled site (see Dataset 2 helps to visualize and perceive additional detail depicted). Lovastatin-lactone had better AutoDock scores and more favorable calculated affinities – despite having fewer hydrogen bond interactions. Both ligands appear to be interacting with ARG-255, ARG-150, and GLN-153, though F420 seems to also interact with ARG-244. F420’s fit is also considerably more constrained, which explains why its AutoDock Vina score is 4.4× worse than lovastatin-lactone’s score.

## Conclusions

Given the large number of ligand-to-site docking scenarios, we were able to observe several key trends that together suggest that statin binding is likely for the two key dehydrogenase targets in question **A5UMI1** and **Q02394**. In most cases, the lactone form appears to have preferential binding over the hydroxyacid form and F420. And in many cases, lovastatin/lactone and simvastatin/lactone appear to have preferential binding to even the native ligands found in the PDB templates used to model **Q02394** and **A5UMI1**. While *in vitro* ligand binding experiments were not conducted, the docking simulations suggest that these dehydrogenases in the main methanogenesis pathway may be possible targets. These results are also consistent with those from a recent phase II clinical trial ( NCT02495623 ^35^) with a proprietary, modified-release lovastatin-lactone (SYN-010) in patients with constipation-predominant, irritable bowel syndrome, which showed a reduction in symptoms and breath methane levels compared to placebo. Given that the lactone form seems to preferentially bind, the next stage of the project is to identify molecules with similar features to lovastatin-lactone that also show similar or better receptor-site interaction potential.

## Data availability

The data referenced by this article are under copyright with the following copyright statement: Copyright: © 2016 Muskal SM et al.

Data associated with the article are available under the terms of the Creative Commons Zero "No rights reserved" data waiver (CC0 1.0 Public domain dedication). 

*F1000Research*: Dataset 1. Raw data for ‘Lovastatin lactone may improve irritable bowel syndrome with constipation (IBS-C) by inhibiting enzymes in the archaeal methanogenesis pathway’, 10.5256/f1000research.8406.d117917 ^36^

*Figshare*: Lovastatin-lactone v. F420 in the A5UMI1 site. doi: 10.6084/m9.figshare.3126538 ^37^
