# Supplementary figures and images for: Lovastatin lactone may improve irritable bowel syndrome with constipation (IBS-C) by inhibiting enzymes in the archaeal methanogenesis pathway
Source: F1000Res. 2016 Jun 22;5:606. Originally published 2016 Apr 8. [Version 3] doi: 10.12688/f1000research.8406.3 (PMC4909102; doi:10.12688/f1000research.8406.3)

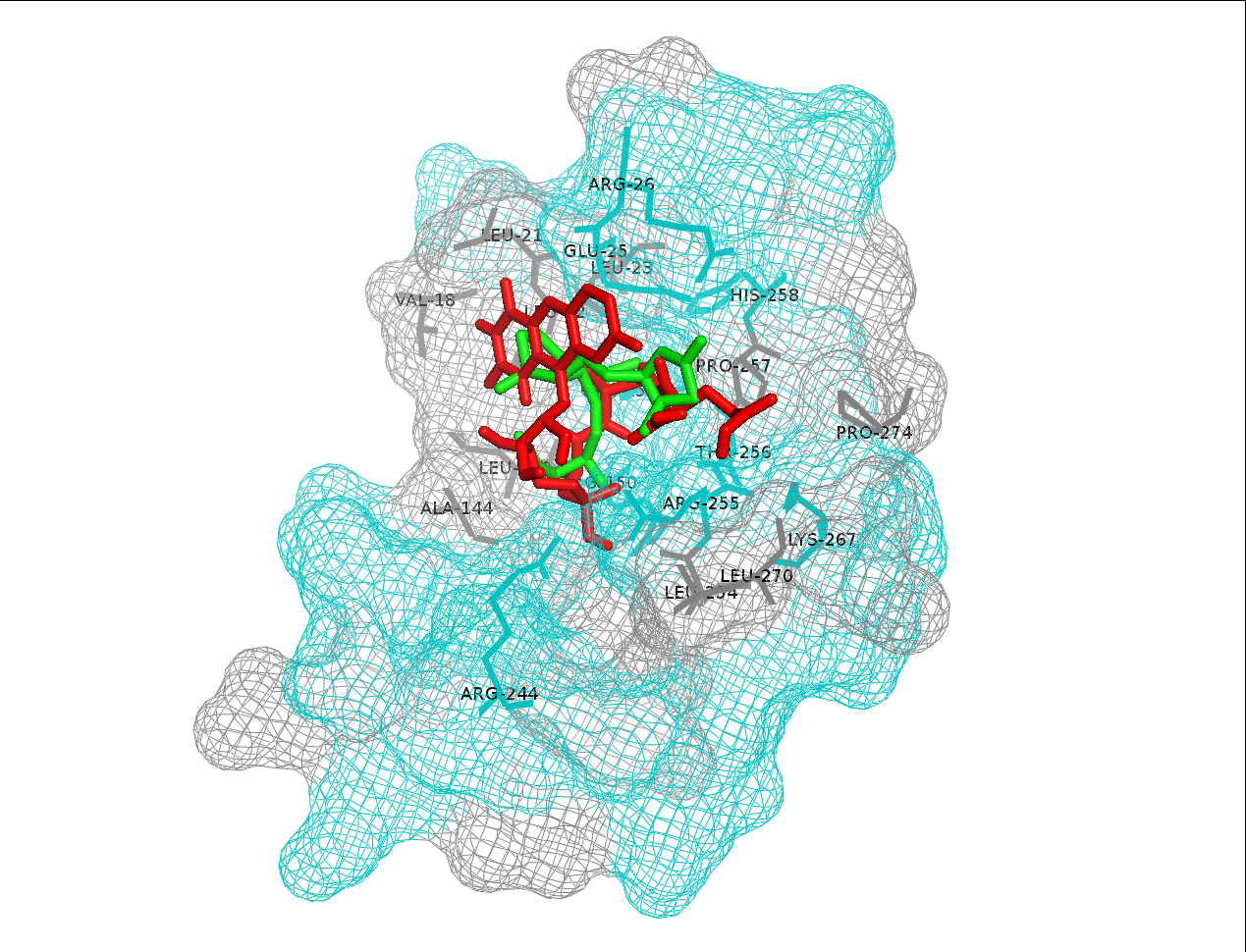

Supplement: Lovastatin-lactone v. F420 in the A5UMI1 site. — The Lovastatin-lactone form is shown with green sticks, and F420 with red sticks. Residues within 5 angstroms of the ligands are labeled and highlighted. Hydrophilic site residues are shown in cyan, and hydrophobic residues in grey. [file f1000research-5-9752-s0000.tgz › gif.gif]
