# Supplementary material for: Lovastatin lactone may improve irritable bowel syndrome with constipation (IBS-C) by inhibiting enzymes in the archaeal methanogenesis pathway
Source: F1000Res. 2016 Jun 22;5:606. Originally published 2016 Apr 8. [Version 3] doi: 10.12688/f1000research.8406.3 (PMC4909102; doi:10.12688/f1000research.8406.3)
Supplement: Raw data for ‘Lovastatin lactone may improve irritable bowel syndrome with constipation (IBS-C) by inhibiting enzymes in the archaeal methanogenesis pathway’ — Includes developed models (pdb format), ligands (mol2 format), sites (pdb format), and vina config files [file f1000research-5-9752-s0001.tgz › 91b3379c-03a9-4f65-98c3-a104fe5c01c7_F1000-Lovastatin.zip!/predockedLigands/PubChemStructures.rtfd/TXT.rtf]

Pasted Graphic.tiff ¬Pasted Graphic 2.tiff ¬Pasted Graphic 3.tiff ¬

Lovastatin Acid


Pasted Graphic 4.tiff ¬
Pasted Graphic 5.tiff ¬
I
PDB

Pasted Graphic 6.tiff ¬Pasted Graphic 7.tiff ¬Pasted Graphic 8.tiff ¬Pasted Graphic 9.tiff ¬Pasted Graphic 10.tiff ¬Pasted Graphic 11.tiff ¬Pasted Graphic 12.tiff ¬Pasted Graphic 13.tiff ¬Pasted Graphic 14.tiff ¬
