# Supplementary material for: Lovastatin lactone may improve irritable bowel syndrome with constipation (IBS-C) by inhibiting enzymes in the archaeal methanogenesis pathway
Source: F1000Res. 2016 Jun 22;5:606. Originally published 2016 Apr 8. [Version 3] doi: 10.12688/f1000research.8406.3 (PMC4909102; doi:10.12688/f1000research.8406.3)
Supplement: Peer review history

# Peer review: Referee response for version 3

**DOI:** 10.5256/f1000research.9752.r14512

**Published:** 2016-06-23

**Authors:** Dušica Vidović

**Affiliations:** 1 Center for Computational Science, University of Miami, Miami, FL, USA

The authors have responded to two questions from my previous report. One question was related to the choice of the docking program AutoDock Vina, while the other question was related to the target of SYN-010 in the clinical trial that authors referenced.

 The authors have justified the use of AutoDock Vina as a well tested docking program that has been found to be "a strong competitor against other programs, and at the top of the pack in many cases".

 Regarding the target of SYN-010 in the clinical trial, although the binding assays for the key dehydrogenases targets were not conducted, the presented docking study of lactone statins generally show preferential docking as compared to F420 (known to interact with both dehydrogenases targets). Based on these findings, the authors suggest that these targets may interact with the lactone form of the statins.

I have read this submission. I believe that I have an appropriate level of expertise to confirm that it is of an acceptable scientific standard.

# Peer review: Referee response for version 2

**DOI:** 10.5256/f1000research.9304.r14327

**Published:** 2016-06-13

**Authors:** Obdulia Rabal

**Affiliations:** 1 Small Molecule Discovery platform, Molecular Therapeutics Program, Center for Applied Medical Research, Navarra's Health Research Institute (IDISNA), University of Navarra, Pamplona, Spain

This paper provides insight on how statins inhibit methane production by direct inhibition of dehydrogenases using modelling studies. Previous work in this field (reference 14) focused on the F420-dependent oxidoreductase. Here, authors explore the potential binding of lovastatin and simvastatin into two F420-dependent methylenetetrahydromethanopterin dehydrogenases (mtd) of *M. smithii* and *Methanopyrus kandleri*. From modelling perspective, the paper is well-structured and it provides enough information to reproduce the results, highlighting common problems involving modelling techniques (e.g. different ligand structures in databases). Considerable computational effort was put into identifying the most probable binding site as well as the active form (lactone versus hydroxyacid) of the ligands. Maybe the major drawback is the lack of *in vitro *results confirming the results, what currently seems difficult to achieve on the basis of the previous authors’ reply. Results from the clinical trial do not fully ensure that the mechanism goes via these targets, so I would suggest putting this correlation/clinical trial into context. Apart from that, I understand that *in vitro *validation of all modelling results is not always possible and the authors use a proper tone to expose their conclusions.

 As a minor point, some comments (e.g. target description) on the PDB entries used to model Q02394 (page 4) would be useful.

I have read this submission. I believe that I have an appropriate level of expertise to confirm that it is of an acceptable scientific standard.

# Peer review: Referee response for version 2

**DOI:** 10.5256/f1000research.9304.r13719

**Published:** 2016-06-07

**Authors:** Dušica Vidović

**Affiliations:** 1 Center for Computational Science, University of Miami, Miami, FL, USA

The authors modeled the structure of F420-dependent methylenetetrahydromethanopterin dehydrogenase (mtd) and systematically predicted possible binding sites in order to test docking of lactone form of statins vs. β-hydroxyacid form of statins, as well as the native ligand F420-coenzyme and other co-complexed ligands found in related structures. They used AutoDock Vina for docking and scoring and their results suggest that: for each modeled site the lactone form of the statins had more favorable site interactions compared to F420; the statin lactone forms generally had the most favorable docking scores, even relative to the native template PDB ligands; and the statin β-hydroxyacid forms had less favorable docking scores.

 Can the authors explain why would the lactone form of lovastatin be a privileged ligand for the predicted binding sites when compared to the other tested ligands? Did the authors try to use another docking program to reproduce these findings?

 Authors also suggest that the lactone form of lovastatin could inhibit the activity of the key M. smithii methanogenesis enzyme mtd in vivo. This is in agreement with the phase II clinical trial (NCT0249562335) the authors refer to. The clinical trial showed a reduction in symptoms and breath methane levels in patients treated with lovastatin-lactone (SYN-010) when compared to placebo. However, it is not clear from the clinical trial reference if SYN-010 inhibits mtd. It would be beneficial for this manuscript to add a reference that indicates the target of SYN-010.

I have read this submission. I believe that I have an appropriate level of expertise to confirm that it is of an acceptable scientific standard, however I have significant reservations, as outlined above.

# Response

**Published:** 2016-06-07

**Authors:** Steven Muskal

**Affiliations:** Eidogen-Sertanty, Inc., USA

Thank you for your comments and questions.

 The key dehydrogenases targets considered in this work [ **A5UMI1 and Q02394**] both interact with F420. However, *in vitro *statin lactone v. F420 binding experiments for these targets were not conducted. In the absence of this data, our modeling and subsequent docking of the lactone statins generally show preferential docking as compared to F420 - which suggests these targets may interact with the lactone form of the statins.

 We selected AutoDock Vina not only because it is open-source and readily available to other researchers, but also because it has has been well tested and found to be "a strong competitor against other programs, and at the top of the pack in many cases" (see http://vina.scripps.edu/index.html). Researchers with access to commercial docking packages are welcome to compare the AutoDock Vina results and data provided with this paper.

 Indeed, Synthetic Biologics' SYN-010 lowered breath methane and improved stool frequency in a Phase 2 Four Week Study in Patients with Irritable Bowel Syndrome with Constipation (IBS-C). This Phase 2 study did not identify the specific ligand-target interaction. However, work by Marsh *et al. *- Lovastatin Lactone Inhibits Methane Production in Human Stool Homogenates ( http://content.stockpr.com/syntheticbiologics/db/220/608/file/Lovastatin+in+Human+Stool-FINAL+Poster+%28ACG+2015%29.pdf) does shed some light. While a messy culture, *M. smithii *was the predominant methanogen in the stool. In this work, lovastatin lactone was identified as the only effective methane inhibitor. Considering other mechanisms by which statins may inhibit methanogenesis, we suggested the dehydrogenases in the main methanogenesis pathway as possibilities.

# Peer review: Referee response for version 1

**DOI:** 10.5256/f1000research.9043.r13313

**Published:** 2016-04-11

**Authors:** Rolf Thauer

**Affiliations:** 1 Max Planck Institute for Terrestrial Microbiology, Marburg, Germany

The manuscript describes modeling studies suggesting that Lovastin lactone, a statin,  inhibits growth of methane-forming archaeon Methanobrevibacter smithii by inhibiting an F420 dependent-enzyme involved in CO2 reduction to methane, namely F420-dependent methylene-tetrahydromethanopterin dehydrogenase (Mtd). Modeling studies had previously indicated that another F420-dependent enzyme, F420H2:NADP oxidoreductase in methanogens could be a site of inhibition (reference 14). The results are interesting, however, before indexing the following information has to be added:

The authors must provide experimental evidence that their theoretical prediction is correct. Show that Lovastin inhibits methane formation from H2 and CO2 in non-growing cell suspensions of M. smithii and/or that Lovastin inhibits Mtd activity in cell extracts of M. smithii of better with the purified enzyme. The authors might want to team up with a lab experienced in the proposed experiments.

Without these experimental data the manuscript would contain nothing really new relative to the results published in reference 14.

The authors must clearly indicate that crystal structures of Mtd with and without substrates bound have been published and give reference to these publications. To only refer to PDBs without the enzyme name is not fair. To indicate in the Abstract, Methods, that there is “no tertiary protein structural information” is more than misleading and can be misunderstood.

I have read this submission. I believe that I have an appropriate level of expertise to confirm that it is of an acceptable scientific standard, however I have significant reservations, as outlined above.

# Response

**Published:** 2016-04-15

**Authors:** Steven Muskal

**Affiliations:** Eidogen-Sertanty, Inc., USA

Thank you for your review and comments.

Based on your comments we fixed the misleading text in the Abstract that you noted, and also corrected a typographical swapping of the sequence IDs in the introduction.

We originally did not feel it necessary to mention all the possible templates considered in the modeling effort, but appreciate the value of doing so. Per your suggestion, we inserted additional text in the modeling section which refers to other relevant PDB templates - e.g. 1QV9 and 1U6I,J,K.  1QV9 - Coenzyme F420-dependent methylenetetrahydromethanopterin dehydrogenase (Mtd) from Methanopyrus kandleri: A methanogenic enzyme with an unusual quaternary structure, and 1U6I,J,K - The structures of native coenzyme F420-dependent methylenetetrahydromethanopterin dehydrogenase at various resolutions, and TLS refinement of the structure of Se-methionine labelled Coenzyme f420-dependent methylenetetrahydromethanopterin dehydrogenase (MTD) from Methanopyrus kandleri. 

With respect to the experimental data comment, while this paper is a computational chemistry paper, we did note and reference the recently completed clinical trial - A Study of the Effect of SYN-010 on Subjects With IBS-C ( https://clinicaltrials.gov/ct2/show/NCT02495623) as well as experimental work by Marsh et al. - Lovastatin Lactone Inhibits Methane Production in Human Stool Homogenates ( http://content.stockpr.com/syntheticbiologics/db/220/608/file/Lovastatin+in+Human+Stool-FINAL+Poster+%28ACG+2015%29.pdf). In the latter, albeit a messy culture, M. smithii was the predominant methanogen in the stool, though the less common Methanosphaera stadtmanae may have been present as well.
